# Medical Writers Good Practitioners

**Published:** 1843-08

**Authors:** 


*Medical Writers Good Practitioners.*—We copy the following pleasant article from the *Boston Journal.* The unjust and illiberal notion which the writer combats is a very common one, and is encountered in the every-day walks of the physician. A still greater prejudice perhaps exists against the physician who cultivates some one or more of the natural sciences. The chemist, the botanist, or the mineralogist, seldom enjoys much patronage as a practitioner. To some extent there is a reason for it, as chemistry, botany, mineralogy and the kindred pursuits are so seductive, that he who becomes addicted to them is apt to *neglect* his professional avocations. But to the article:

An idea is prevalent in the world, and even fostered among medical men, perhaps from interested motives, that medical writers are not generally the best practitioners. The very reverse of this is more generally the fact. The nature of their researches obliges them to became familiar with the opinions and practice of the best practitioners, and to treasure them up with the most scrupulous attention. A store of useful and practical information is always at hand, which is ever ready to be applied to the cases under consideration and treatment. Writers, too, always provide themselves with large and respectable libraries for the purpose of investigating all the subjects on which they treat, as well as for the intrinsic value of the information they obtain from the perusal of them. Dr. Rush observes, "If a physician obtain skill by his own solitary experience, how much more will he acquire by availing himself of the experience of several hundred physicians, which he can only obtain by availing himself of the opportunity of perusing a large medical library." And if it be true, which can admit of but little doubt, that a physician cannot accurately remember the details of his practice more than three years, of how much importance is it that he should be in the habit of recording all important facts and cases which may occur to his notice and observation, and be continually treasuring up fresh stores of knowledge by unwearied attention to books. A physician's studies are never finished till the close of life. I was always very much pleased with the following anecdote of Dr. Rush. "As two young physicians were once conversing in his presence, one of them said—'When I *finished* my studies.' When *you* finished *your* studies!' said the doctor, abruptly; 'why you must be a happy man to have finished your studies so young! I do not expect to finish *mine* while I live.' "

Notwithstanding the above observations, we have too much reason to believe that a great proportion of practising physicians in America do not devote much of their time and attention to study after they commence the practice of their profession; and it is from this mental idleness, as Dr. Rush observes, that "it is no uncommon thing for an old physician (from his neglect of books) to be more ignorant than he was when he commenced the practice of his profession."

Let it not be said that a physician has not time to record his experience or even to read. Some of the most extensive practitioners have been the most voluminous writers, and most industrious readers. In proof of this, we need only to mention the example of some of the fathers of our profession—such as Hippocrates, Galen, Celsus, Hoffman, Sydenham, Boerhaave, Van Swieten, Wistar, the Hunters, Monro, Cullen, and a host of others, almost all of whom were engaged in the most extensive and most lucrative practice, and were also authors of several most voluminous works. Dr. Good was one of our most elaborate writers. He has written several works, besides his great one on the practice of physic, yet the income of his practice was seven or eight thousand dollars a year. Sir Astley Cooper more than doubled the amount charged by Dr. Good in the same space of time; and yet, he was continually furnishing the world, through the medium of his writings, with the result of his knowledge and experience. Innumerable other examples in Europe might be mentioned of a similar nature. In fact, the best and most learned writers there, were altogether the best and most successful practitioners. Dr. Clark and Sir William Jones may be added to this list. They never for a moment neglected the duties of their profession. Indeed they excelled in the practice of that profession, and they were among the most eminent in Europe, in science and literature.

Our own country, too, is rich in examples to prove that our best practitioners are, and ever have been, our ablest writers. Among our deceased medical men we need only mention the immortal names of Rush, Barton, Redman, Wistar, Dorsey, Dewees, Physick, Parish, Ramsay, Miller, Hosack, Godman, Eberle, Warren, Gorham, and numerous others, whose writings alone would fill a decent library. Among the living we take great pleasure in enumerating the names of Jackson, Warren, Bigelow, Hale, Ware, Hayward, Shattuck, Smith, Holmes, Woodward, and many others in Massachusetts; of Parsons, Senter and others, in Rhode Island; of Tully, Ives, Sumner and others, in Connecticut; of Mott, Beck, McNaughton, Reese, Paine, Lee, Forrey, the Smiths, Delafield, *cum multis aliis*, in New York; of the venerable and learned Coxe, Samuel Jackson, Hays, Gerhard, the McClellans, Horner, Gibson, Bache, Dunglison, Wood, Bell, and innumerable others, in Philadelphia; not forgetting the names of N. R. Smith, Annan, Sewall, and others, at the South; and Gross, Drake, Cartwright, Mussey, Hildreth and Kirtland, beyond the Alleghanies. Let no one accuse me of partiality in this enumeration. I am sensible that I have omitted the names of very many who have been equally successful with their pens, and in their practice. My object was not to give a list of our celebrated writers, for that would fill a sheet, but to mention a few which, without much reflection, presented themselves to my mind in illustration of the truth of the opinion that our ablest and most elaborate medical writers are also our *very best practitioners*. W. W.

January 8th, 1843.

